# EARLY LIFE COGNITION, EDUCATION, AND DEMENTIA RISK

**DOI:** 10.1093/geroni/igad104.0002

**Published:** 2023-12-21

**Authors:** Pamela Herd, Victoria Williams, Kamil Sicinski

**Affiliations:** Georgetown University, Washington, District of Columbia, United States; University of Wisconsin-Madison, Madison, Wisconsin, United States; University of Wisconsin-Madison, Madison, Wisconsin, United States

## Abstract

Educational attainment is one of the strongest predictors of Alzheimer’s and Related Dementias (ADRD). Yet, much remains unknown as to how early life factors, particularly early life cognitive function, and early life environments, such as family environments, help explain why education so robustly protects against later life dementia. Is the influence of educational attainment not so much the additional schooling, but rather antecedent factors, like early life cognition and family environments that both build cognitive reserve and predict subsequent educational attainment? In short, is education merely a proxy for these antecedent influences? Consequently, we use the Wisconsin Longitudinal Study, which includes 80 years of prospectively collected data on a sample of 1 in every 3 high school graduates, and a selected sibling, from the class of 1957. Sibling models, and the inclusion of prospectively collected early life cognitive function and educational histories, allows us to explore the role of these early life conditions and the role they play in shaping the relationship between education and ADRD. We find a robust independent influence of education on ADRD risk, early life family environments had little impact on the relationship. Adolescent cognition, however, which we show is influenced by family environments, does partly explain why educational attainment influences later life ADRD risk, though both adolescent IQ and education independently influence the risk for and resilience against dementia.

